# Silencing a Simple Extracellular Leucine-Rich Repeat Gene *OsI-BAK1* Enhances the Resistance of Rice to Brown Planthopper *Nilaparvata lugens*

**DOI:** 10.3390/ijms222212182

**Published:** 2021-11-10

**Authors:** Miaofen Ye, Peng Kuai, Shuting Chen, Na Lin, Meng Ye, Lingfei Hu, Yonggen Lou

**Affiliations:** 1 State Key Laboratory of Rice Biology & Ministry of Agriculture Key Lab of Agricultural Entomology, Institute of Insect Sciences, Zhejiang University, Hangzhou 310058, China; 11616070@zju.edu.cn (M.Y.); kpchen7493@163.com (P.K.); shut_chen@163.com (S.C.); lin_na@zju.edu.cn (N.L.); mengye@tricaas.com (M.Y.); lingfeihu@zju.edu.cn (L.H.); 2 Hainan Institute, Zhejiang University, Sanya 572025, China

**Keywords:** rice, *OsI-BAK1*, *Nilaparvata lugens*, ethylene, plant defense responses, simple extracellular leucine-rich repeat protein

## Abstract

Many plant proteins with extracellular leucine-rich repeat (eLRR) domains play an important role in plant immunity. However, the role of one class of eLRR plant proteins—the simple eLRR proteins—in plant defenses against herbivores remains largely unknown. Here, we found that a simple eLRR protein OsI-BAK1 in rice localizes to the plasma membrane. Its expression was induced by mechanical wounding, the infestation of gravid females of brown planthopper (BPH) *Nilaparvata lugens* or white-backed planthopper *Sogatella furcifera* and treatment with methyl jasmonate or abscisic acid. Silencing *OsI-BAK1* (ir-*ibak1*) in rice enhanced the BPH-induced transcript levels of three defense-related WRKY genes (*OsWRKY24*, *OsWRKY53* and *OsWRKY70*) but decreased the induced levels of ethylene. Bioassays revealed that the hatching rate was significantly lower in BPH eggs laid on ir-*ibak1* plants than wild-type (WT) plants; moreover, gravid BPH females preferred to oviposit on WT plants over ir-*ibak1* plants. The exogenous application of ethephon on ir-*ibak1* plants eliminated the BPH oviposition preference between WT and ir-*ibak1* plants but had no effect on the hatching rate of BPH eggs. These findings suggest that OsI-BAK1 acts as a negative modulator of defense responses in rice to BPH and that BPH might exploit this modulator for its own benefit.

## 1. Introduction

When attacked by herbivorous insects, plants recognize patterns associated with damage and with herbivores via pattern recognition receptors (PRRs) and respond by activating a series of early signaling events: increasing cytosolic Ca^2+^ concentrations, triggering a burst of reactive oxygen species (ROS) and activating mitogen-activated protein kinase (MPK) cascades [1,2]. These early events trigger signaling pathways mediated by phytohormones consisting mainly of jasmonic acid (JA), ethylene (ET) and salicylic acid (SA), which reorganize the transcriptome, proteome and metabolome, respectively, of the plant and thereby enhance plants’ resistance to herbivores [3,4,5,6].

Proteins with extracellular leucin-rich repeat (eLRR) domains are categorized into five classes, including polygalacturonase inhibitor protein (PGIP)-like proteins, leucine-rich repeat extensin-like proteins, leucine-rich repeat receptor-like proteins (LRR-RLPs), leucine-rich repeat receptor-like kinases (LRR-RLKs) and simple eLRR domain proteins [7,8,9]. Many among the first four classes of eLRR proteins, especially LRR-RLKs and LRR-RLPs, have been reported to play an important role in defense responses of plants to pathogens and herbivores [10,11,12]. In Arabidopsis, for example, flagellin-sensitive 2 (FLS2), an LRR-RLK protein, binds flagellin peptide 22, an elicitor secreted by bacteria [13,14], and interacts with another LRR-RLK, brassinosteroid insensitive 1–associated kinase 1 (BAK1), to form a PRR complex, the FLS2-BAK1 complex [15]. The complex activates downstream of defense-related signaling cascades and regulates plant defenses to pathogens [16,17]. In rice, an LRR-RLK, Xanthomonas resistance 21 (XA21), recognizes tyrosine-sulfated protein RaxX (required for activating XA21-mediated immunity X) from *Xanthomonas oryzae* pv. *oryzae* (*Xoo*) and then induces the production of ROS and ethylene and enhances the resistance of rice to *Xoo* [18,19,20,21]. A rice LRR-RLK, OsLRR-RLK1, acts upstream of the MPK cascades and positively regulates both the biosynthesis of herbivore-induced JA and ethylene and plants’ resistance to striped stem borer (SSB) *Chilo suppressalis* [22]. In cowpeas (*Vigna unguiculata*), an LRR-RLP has been reported to function as a receptor of inceptins, elicitors that exist in the oral secretions of many Lepidopteran species; this receptor is sufficient to confer inceptin-induced responses and enhances the defense responses against armyworms *Spodoptera exigua* [23]. Moreover, the fitness of leaf beetles *Phaedon cochleariae* fed on PGIP (*AtPGIP1* or *AtPGIP2*)-deficient plants is higher than the fitness of those fed on wild-type (WT) plants [24].

Simple eLRR proteins have also been reported to regulate the defense responses of plants to pathogens. For example, some simple eLRR genes, such as *SlLRP* in *Solanum lycopersicum* [25], *SbLRR* and *SbLRR2* in *Sorghum bicolor* [26,27] and *NtLRP1* and *NtLRR1* in *Nicotiana tabacum* [28,29], are responsive to pathogen infections. In pepper *Capsicum annuum*, a simple eLRR protein, CaLRR1, interacts with a hypersensitive-induced reaction (HIR) protein, CaHIR1, and two pathogenesis-related proteins, CaPR10 and CaPR4b, and then regulates the plant defense responses [30]. A simple eLRR protein in rice, OsLRR1, whose transcript levels are induced by pathogen infection, wounding and treatment with SA or JA, enters the endosomal pathway and interacts with plasma membrane-localized OsHIR1; the ectopic expression of OsLRR1 in *Arabidopsis thaliana* enhances the resistance of plants to *Pseudomonas syringae* pv. *tomato* DC3000 (*Pst* DC3000) by increasing the transcript levels of the defense genes related to the SA (*AtPR1* and *AtPR2*)- and JA (*AtPDF1.2*)-mediated pathways [7,9]. Expressing OsLRR1 in Chinese cabbage *Brassica rapa* L. subsp. *pekinensis* enhances plants’ resistance to bacterial soft rot by activating defense-related genes (*PDF1*, *BrPGIP2*, glucanase genes *MERI5B* and *Meri-5* and hypersensitive cell death marker gene *Athsr3*) [7]. Moreover, OsLRR1 is required for XA21-mediated rice defense responses to *Xoo* [31]. However, whether and how simple eLRR proteins regulate plants’ defenses to herbivores remains largely unknown.

Rice (*Oryza sativa* L.), one of the most important food crops in the world, suffers heavily from predation by many herbivorous insects. Brown planthopper (BPH) *Nilaparvata lugens* (Stål), a major hemipteran insect pest, damages rice mainly by feeding on phloem sap, laying eggs in plant tissues and transmitting viruses [32]. Previous studies have revealed that, when rice plants are infested by BPH, they respond by activating a complex signaling network, primarily MPK cascades and pathways mediated by JA, SA, ethylene and hydrogen peroxide (H_2_O_2_) [33,34,35,36,37,38]; the defense responses, such as the accumulation of trypsin proteinase inhibitors (TrypPIs) and the release of herbivore-induced plant volatiles (HIPVs), may influence the performance of conspecific and non-conspecific herbivores directly, as well as indirectly, by regulating the role of the natural enemies of herbivores [22,36,37,38,39,40,41,42]. Although some eLRR proteins play an important role in regulating rice plants’ defense responses to pathogens and herbivores, as stated above, whether simple eLRR proteins are involved in the defense responses of rice to herbivores remains unclear.

In this study, we identified an herbivore-induced simple eLRR protein gene in rice, *OsI-BAK1*, which responds to infestation by gravid BPH females [41]. To investigate its role in BPH-induced defenses in rice, we obtained stably transformed rice plants rendered deficient in *OsI-BAK1* by RNA interference (RNAi). Combining molecular biology, phytohormone analyses and bioassays, we demonstrate that OsI-BAK1 positively regulates the biosynthesis of BPH-induced ET and decreases the resistance of rice to BPH.

## 2. Results

### 2.1. Isolation and Characterization of OsI-BAK1

**Figure 1:**
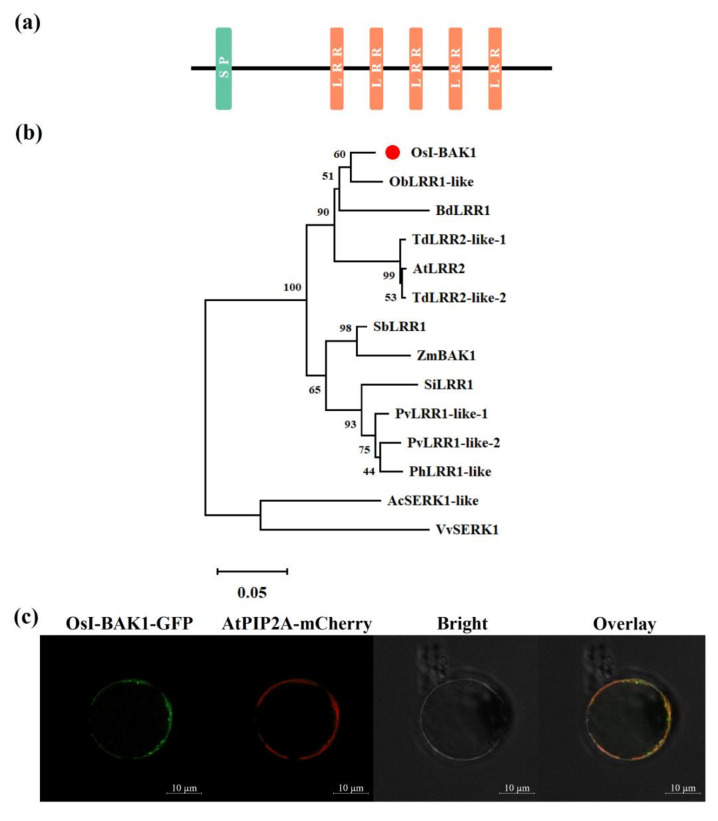
Structure, phylogenetic relationship and subcellular localization of OsI-BAK1. (**a**) Schematic structure of OsI-BAK1. SP, signal peptide; LRR, leucin-rich repeat. (**b**) Sequence alignment of OsI-BAK1 and its homologs. Species abbreviations are included before the protein names: Ac, *Ananas comosus*; At, *Aegilops tauschii* subsp. *strangulate*; Bd, *Brachypodium distachyon*; Ob, *Oryza brachyantha*; Os, *Oryza sative*; Ph, *Panicum hallii*; Pv, *Panicum virgatum*; Sb, *Sorghum bicolor*; Si, *Setaria italica*; Td, *Triticum dicoccoides*; Vv, *Vitis vinifera* and Zm, *Zea mays*. Plant species and accession numbers from the NCBI database are as follows: AcSERK1-like, XP_020105022.1; AtLRR2, XP_020193068.1; BdLRR1, XP_003562364.1; ObLRR1-like, XP_006650233.1; OsI-BAK1, XP_015631230.1; PhLRR1-like, XP_025793013.1; PvLRR1-like-1, XP_039825633.1; PvLRR1-like-2, XP_039785598.1; SbLRR1, XP_002467662.1; SiLRR1, XP_004983941.1; TdLRR2-like-1, XP_037465236.1; TdLRR2-like-2, XP_037458478.1; VvSERK1, XP_002263235.1 and ZmBAK1, NP_001148919.1. The red dot indicates OsI-BAK1. The scale bar represents 0.05 amino acid substitution per site in the primary structure. (**c**) Subcellular localization of OsI-BAK1. Polyethylene glycol-mediated transient expression in rice protoplasts of OsI-BAK1-GFP and AtPIP2A-mCherry. OsI-BAK1-GFP, green fluorescent protein (GFP) fluorescence from OsI-BAK1-GFP; AtPIP2A-mCherry, mCherry fluorescence from AtPIP2A-mCherry; Bright, bright field and Overlay, co-localization of the OsI-BAK1-GFP and AtPIP2A-mCherry proteins. Bars, 10 μm.

The transcriptome data from rice plants infested by gravid females of BPH showed that the transcript level of a simple eLRR gene was significantly upregulated [41]. By cloning this gene, we found that its sequence was 100% identical to the previously reported *OsI-BAK1* (brassinosteroid insensitive 1-associated kinase 1) [43]. The sequence analysis found that *OsI-BAK1* includes an open reading frame of 657 bp; this frame, which encodes a protein of 219 amino acids with a predicted molecular weight of 23.64 kDa, contains a signal peptide and five LRR motifs (Figure 1a and Figure S3). BLASTP searches in the NCBI showed that OsI-BAK1 shares a high similarity with simple eLRR proteins, such as ObLRR1-like (96.92% identity) in *Oryza brachyantha*, BdLRR1 (94.76%) in *Brachypodium distachyon*, AtLRR2 (94.76%) in *Aegilops tauschii* subsp. *Strangulate* and TdLRR2-like-1 (87.61%) and TdLRR2-like-2 (87.61%) in *Triticum dicoccoides* (Figure 1b).

A subcellular localization assay using a fusion gene (*OsI-BAK1* with green fluorescent protein) showed that the fluorescent signal emitted from the fusion protein was observed on the plasma membrane; this signal overlapped with the fluorescent signal emitted from the membrane-localized positive control protein AtPIP2A (Figure 1c). The results suggest that OsI-BAK1 was a plasma membrane-localized eLRR protein and may play a role in signal recognition and transduction.

### 2.2. Herbivore Infestation, Wounding and JA and Abscisic Acid Treatment Enhance Transcript Levels of OsI-BAK1

**Figure 2:**
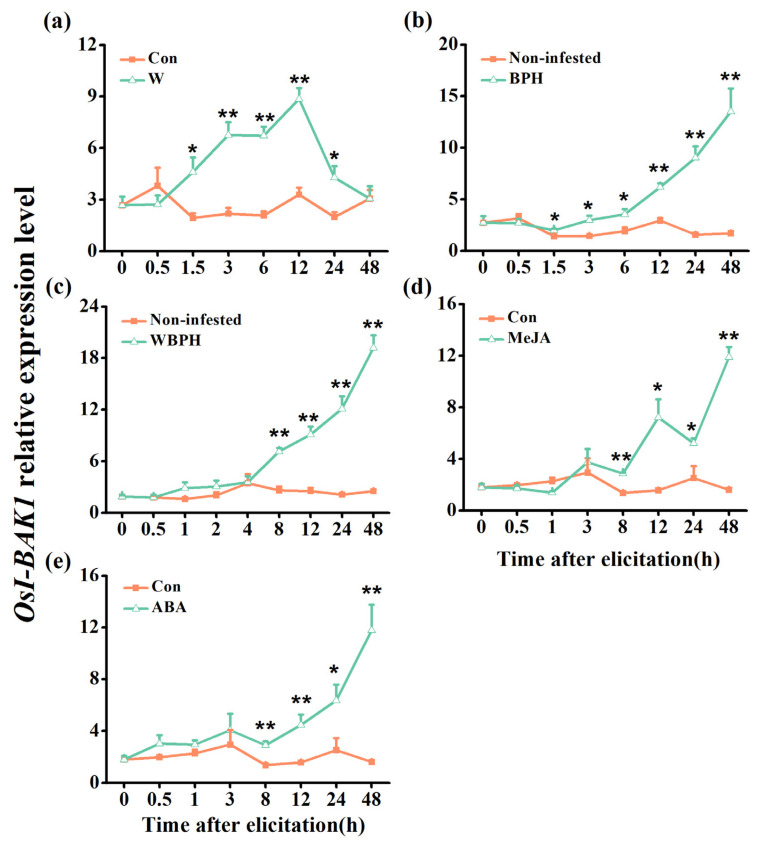
Relative expression levels of *OsI-BAK1* in rice leaf sheaths after different treatments. Mean transcript levels (+SE, n = 5) of *OsI-BAK1* in rice leaf sheaths that were mechanically wounded (W, (**a**)), infested with gravid brown planthopper (BPH) females (**b**), gravid white-backed planthopper (WBPH) females (**c**) or treated with methyl jasmonate (MeJA) (**d**) or abscisic acid (ABA) (**e**). Non-infested, plants that were covered in an empty glass cylinder without BPH or WBPH; Con, control plants corresponding to mechanical wounding or MeJA or ABA treatment. Asterisks represent significant differences between the treatments and controls at each time point (* *p* < 0.05 and ** *p* < 0.01; Student’s *t*-tests).

Compared with the corresponding controls, all of the measured treatments—BPH infestation, white-backed planthopper (WBPH) infestation, mechanical wounding and treatment with methyl jasmonate (MeJA) or abscisic acid (ABA)—enhanced the transcript level of *OsI-BAK1* in plants (Figure 2). Unlike BPH infestation and mechanical wounding, both of which induced the expression of *OsI-BAK1* quickly starting 1.5 h after treatment (Figure 2a,b), the other three treatments induced the expression of *OsI-BAK1* relatively later, starting 8 h after treatment (Figure 2c–e). Moreover, the effect of mechanical wounding on the expression of *OsI-BAK1* lasted for less time, peaking at 12 h and returning to control levels at 48 h, compared to the effect of other treatments on the expression of *OsI-BAK1* (in these, the expression of *OsI-BAK1* peaked 48 h after treatment).

### 2.3. Silencing OsI-BAK1 in Rice

**Figure 3:**
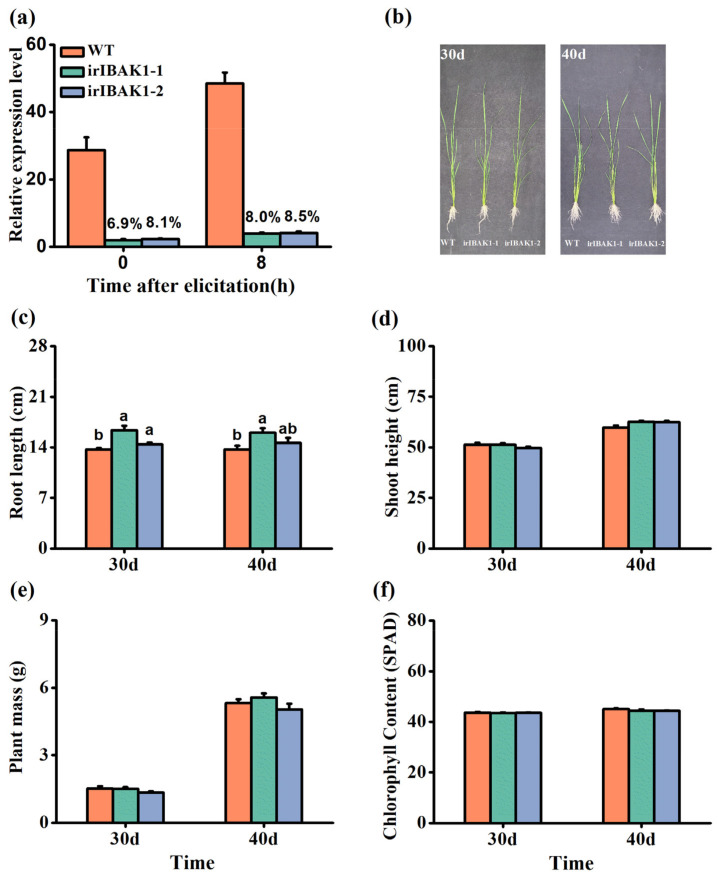
Transgenic lines silencing *OsI-BAK1* and their growth phenotypes. (**a**) Mean expression levels (+SE, n = 5) of *OsI-BAK1* in wild-type (WT) and ir-*ibak1* plants at 0 and 8 h after BPH infestation. Percentages indicate the percentage of the expression levels of *OsI-BAK1* in ir-*ibak1* plants compared to the expression levels of *OsI-BAK1* in WT plants. (**b**) The growth phenotype of 30- and 40-day-old WT and ir-*ibak1* plants in the greenhouse. (**c**–**f**) Mean root length (**c**), shoot height (**d**), plant mass (**e**) and chlorophyll content (**f**) (+SE, n = 10) of WT and ir-*ibak1* plants at 30 or 40 days old in the greenhouse. Different letters represent significant differences among the lines (*p* < 0.05, Tukey’s HSD post-hoc tests).

To investigate the function of OsI-BAK1 in herbivore-induced defense responses in rice, we obtained two homozygous *OsI-BAK1*-silenced lines (irIBAK1-1 and irIBAK1-2) with a single insertion (Figure S4a, See Supplementary Materials). The transcriptional analysis showed that the transcript level of *OsI-BAK1* in the two ir-*ibak1* lines was only 6.9–8.5% of that in WT plants 0 and 8 h after BPH infestation (Figure 3a).

Based on the database from the Rice Genome Annotation Project (http://rice.plantbiology.msu.edu/index.shtml; accessed on 29 October 2019), five genes shared high similarities in nucleotide sequences with OsI-BAK1: LOC_Os01g59440 (92.31% identity), LOC_Os11g31540 (90.00%), LOC_Os01g17250 (89.51%), LOC_Os11g31530 (89.09%) and LOC_Os05g41230 (82.42%). Among these five genes, two (LOC_Os11g31530 and LOC_Os05g41230) showed such low transcript levels that their transcripts were not detected; the other three genes were not co-silenced by the RNAi construct in the transgenic plants (Figure S4b–d). Therefore, the specificity of the RNAi sequence was high. Compared with WT plants, ir-*ibak1* plants showed only a slight difference in growth phenotypes (Figure 3b): the root length of ir-*ibak1* plants 30 or 40 days old was longer than the root length of WT plants (Figure 3c), whereas no difference was observed in shoot height, plant mass or chlorophyll contents between WT and ir-*ibak1* plants (Figure 3d–f).

### 2.4. OsI-BAK1 Negatively Regulates the Transcript Level of Defense-Related WRKYs

**Figure 4:**
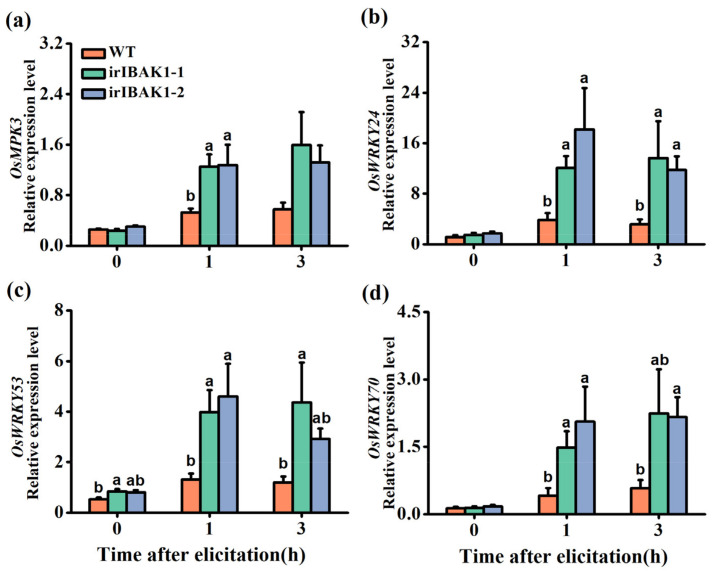
Silencing *OsI-BAK1* enhances the transcript levels of defense-related MPKs and WRKYs. Mean expression levels (+SE, n = 5) of *OsMPK3* (**a**), *OsWRKY24* (**b**), *OsWRKY53* (**c**) and *OsWRKY70* (**d**) in WT and ir-*ibak1* plants after gravid BPH female infestation. Different letters represent significant differences among the lines (*p* < 0.05, Tukey’s HSD post-hoc tests).

MPK cascades and WRKY transcription factors (TFs) are key regulators in plant defense responses [44,45]. Thus, we detected the kinase activity of two MPKs, OsMPK3 and OsMPK6, and the transcript levels of *OsMPK3* and *OsMPK6* [46,47], as well as of three WRKY genes (*OsWRKY24*, *OsWRKY53* and *OsWRKY70*), all of which have been reported to play an important role in the defense responses of plants to pathogens or herbivores [33,34,48,49] in ir-*ibak1* and WT plants. The results showed that the transcript levels of *OsMPK3* were higher in the ir-*ibak1* lines than in WT plants at 1 h after BPH infestation (Figure 4a), whereas no difference was found in the transcript levels of *OsMPK6* between WT and ir-*ibak1* plants before or after BPH infestation (Figure S5a). Although BPH infestation induced the kinase activation of OsMPK3, it did not induce the kinase activation of OsMPK6; no difference in the kinase activation of both OsMPK3 and OsMPK6 was observed between the WT and ir-*ibak1* lines (Figure S5b–d). Silencing *OsI-BAK1* enhanced not only the BPH-induced transcript levels of *OsWRKY24*, *OsWRKY53* and *OsWRKY70* but, also, the constitutive transcript levels of *OsWRKY53* (Figure 4b–d).

### 2.5. Silencing OsI-BAK1 Reduces the Accumulation of BPH-Induced Ethylene

**Figure 5:**
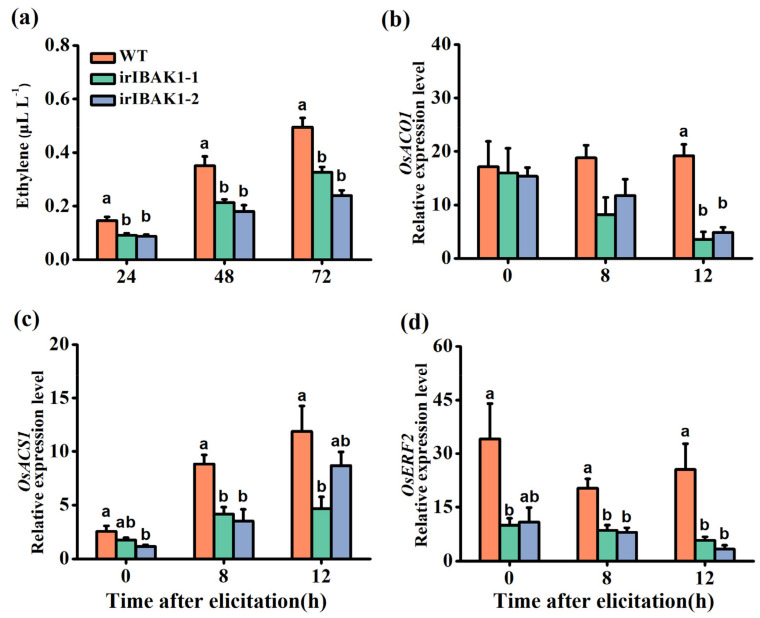
Silencing *OsI-BAK1* decreases the BPH-induced levels of ethylene (ET). (**a**) Mean levels (+SE, n = 8) of ET emitted from individual WT and ir-*ibak1* plants at 24, 48 and 72 h after gravid BPH female infestation. (**b**–**d**) Mean transcript levels (+SE, n = 5) of *OsACO1* (**b**), *OsACS1* (**c**) and *OsERF2* (**d**) in WT and ir-*ibak1* plants at different time points after they were infested by gravid BPH females. Different letters represent significant differences among the lines (*p* < 0.05, Tukey’s HSD post-hoc tests).

The JA-, SA-, ET-, ABA- and H_2_O_2_-mediated signaling pathways regulate the resistance of rice to BPH [36,39,50]. Therefore, we investigated the changes in the concentrations of these six signal molecules in WT and ir-*ibak1* plants before and after they were infested by BPH. A remarkable decrease in the ET levels in transgenic plants compared with WT plants was observed when plants were infested with gravid females of BPH for 24, 48 or 72 h (Figure 5a). Consistently, the transcript levels of two genes related to ET biosynthesis (*OsACO1* and *OsACS1*) [51,52] and of one transcription factor, *OsERF2*, which might be related to the ET pathway [53], decreased in the ir-*ibak1* lines compared with WT plants (Figure 5b–d). However, although BPH induced the accumulation of JA, JA-Ile, ABA and H_2_O_2_ in plants, no difference in the levels of these signal molecules was observed between the WT and ir-*ibak1* plants, except for the constitutive level of H_2_O_2_, which was 1.3-fold higher in irIBAK1-1 plants than in WT plants (Figure S6a–d). Moreover, silencing *OsI-BAK1* did not influence the SA accumulation in plants (Figure S6e).

### 2.6. Silencing OsI-BAK1 Enhances the Resistance of Rice to BPH

**Figure 6:**
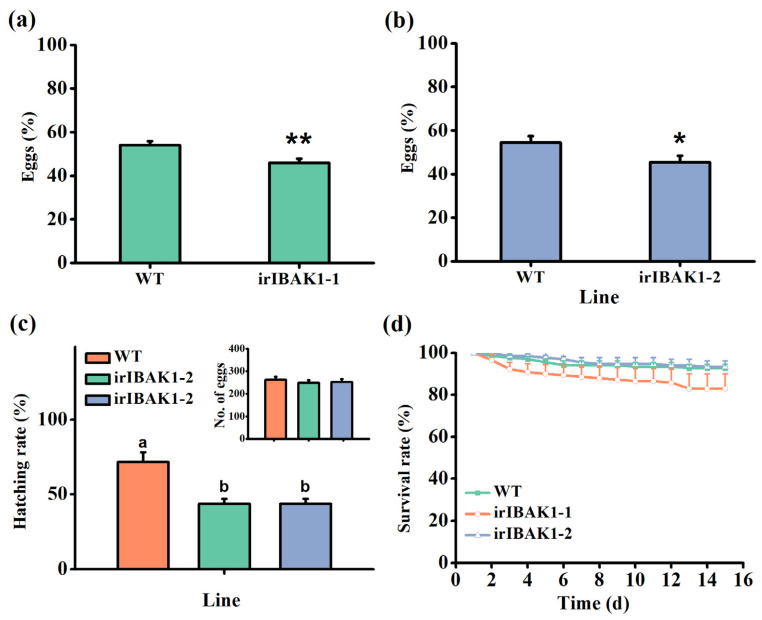
Silencing *OsI-BAK1* enhances the resistance of rice to BPH. (**a**,**b**) Mean percentage (+SE, n = 10) of BPH eggs per plant on pairs of plants (WT versus irIBAK1-1 or irIBAK1-2) 48 h after the release of BPH. Asterisks represent significant differences between the WT and transgenic plants (irIBAK1-1 or irIBAK1-2) (* *p* < 0.05 and ** *p* < 0.01; Student’s *t*-tests). (**c**) Mean hatching rate (+SE, n = 10) of BPH eggs laid on WT and ir-*ibak1* plants. Inserts: mean number of BPH eggs (+SE, n = 10) per plant on WT and ir-*ibak1* plants. Different letters indicate significant differences among the lines (*p* < 0.05, Tukey’s HSD post-hoc tests). (**d**) Mean survival rate (+SE, n = 10) of BPH nymphs on WT and ir-*ibak1* plants 0–16 d after the newly hatched nymphs were placed on the plants.

The bioassays revealed that gravid BPH females preferred to lay eggs on WT plants over ir-*ibak1* plants: the number of eggs laid on irIBAK1-1 and irIBAK1-2 plants was only 66.38% and 66.52% of the number laid on WT plants (Figure 6a,b). Moreover, the hatching rate of BPH eggs laid on ir-*ibak1* plants was significantly lower than that of BPH eggs laid on WT plants (Figure 6c). However, silencing *OsI-BAK1* did not influence the survival rate of BPH nymphs (Figure 6d).

**Figure 7:**
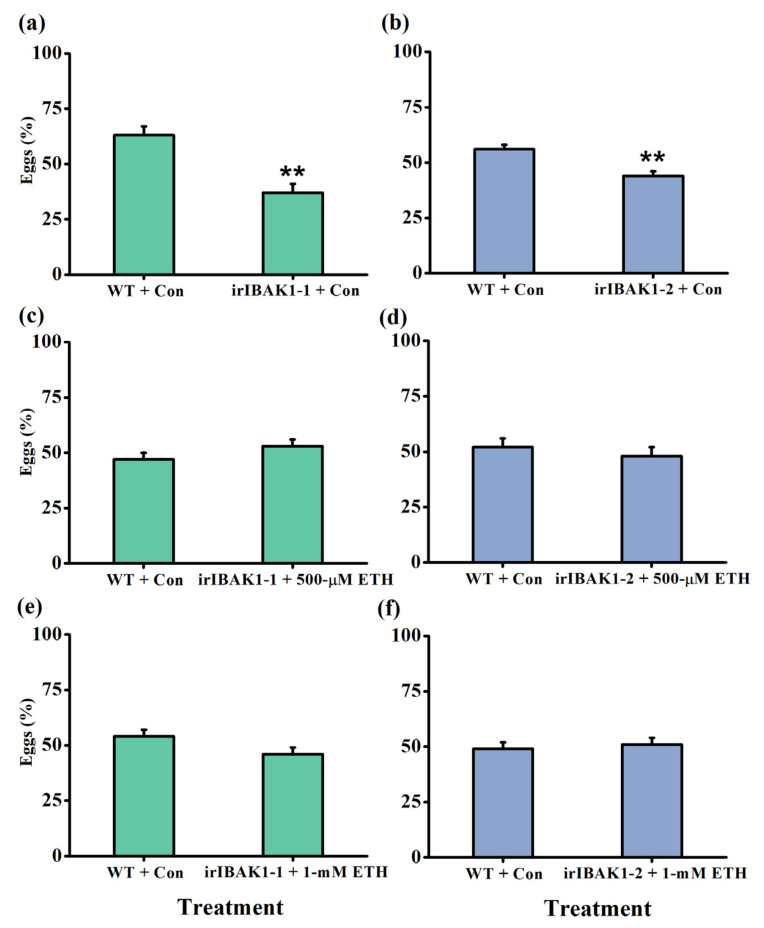
Spraying ethephon abolishes the difference in oviposition preference of BPH for WT and ir-*ibak1* plants. (**a**,**b**) Mean percentage (+SE, n = 10) of BPH eggs per plant on pairs of plants (WT versus irIBAK1-1 or irIBAK1-2) that were sprayed with ddH_2_O (with 0.02% Tween-20) 48 h after the release of BPH. (**c**–**f**) Mean percentage (+SE, n = 10) of BPH eggs per plant on pairs of plants, WT plants that were sprayed with ddH_2_O (with 0.02% Tween-20) versus irIBAK1-1 or irIBAK1-2 plants that were sprayed with 500-μM ethephon (ETH) (**c**,**d**) or WT plants that were sprayed with ddH_2_O (with 0.02% Tween-20) versus irIBAK1-1 or irIBAK1-2 plants that were sprayed with 1-mM ETH (**e**,**f**) 48 h after the release of BPH. Con, control solution. Asterisks represent significant differences between the treated plants and control plants (** *p* < 0.01; Student’s *t*-tests).

To investigate whether the enhanced resistance of ir-*ibak1* plants to BPH was due to their reduced emission of ET, we treated ir-*ibak1* plants by spraying them with ethephon and carried out the bioassays as above. The data showed that the decrease in preference of BPH for ir-*ibak1* plants was abolished when ir-*ibak1* plants were individually sprayed thoroughly with 500-μM or 1-mM ethephon (Figure 7). However, spraying ethephon on ir-*ibak1* plants with the same concentration did not affect the hatching rate of BPH eggs (Figure S7).

## 3. Discussion

Simple eLRR proteins have been reported to play an important role in the defense responses of plants to pathogens by influencing the defense-related signaling pathways [9,30,54]. In this study, we found that a simple eLRR gene in rice, *OsI-BAK1*, is induced by BPH or WBPH infestation, mechanical wounding and treatment with MeJA or ABA. Silencing *OsI-BAK1* increased the transcript levels of defense-related genes *OsMPK3*, *OsWRKY24*, *OsWRKY53* and *OsWRKY70* and decreased the BPH-induced ET levels, which subsequently enhanced the resistance of rice to BPH. These results demonstrate that *OsI-BAK1* functions as an important negative regulator in the defense responses of rice to BPH.

Simple eLRR genes have been reported to exhibit specific expression patterns. For example, some simple eLRR genes, such as *SlLRP* in *Solanum lycopersicum* [25], *SLRR* and *SbLRR2* in *Sorghum bicolor* [26,27] and *OsLRR1* in rice [9], are reportedly induced by pathogen infection. Some simple eLRR genes are responsive to abiotic stresses: *OsLRR1* and *CaLRR1* are responsive to mechanical wounding [9,55], and *NtLRR2* and *CaLRR1* are responsive to salt treatment [29,55]. Moreover, some simple eLRR genes respond differently to phytohormone treatment. In pepper, for example, *CaLRR1* is induced by ABA treatment but not by treatment with SA, MeJA or ET [55], whereas *SbLRR2* is strongly induced by MeJA but not by SA or 1-aminocyclopropane-1-carboxylic acid (ACC) [27]. In addition, the transcript levels of several simple eLRR genes are reported to be tissue-specific. For example, *NtLRR2* is mainly expressed in roots rather than in stems and leaves [29]. *LRRop-1* from *Arabidopsis* is enriched in seeds and rosette leaves [56]. We observed that *OsI-BAK1* was induced by mechanical wounding, infestation with BPH and WBPH and treatment with MeJA and ABA (Figure 2). Together, these data suggest that each simple eLRR gene is expressed in particular tissues and responds differently to biotic and abiotic stresses, thereby playing different roles in plant growth, development and defenses against various stresses.

The subcellular localization of eLRR proteins has been researched extensively. Among these, LRR-RLKs and LRR-RLPs are plasma membrane-localized proteins [57,58], whereas PGIP-like proteins and leucine-rich repeat extensin-like proteins are cell wall-localized [59,60]. The subcellular localization of simple eLRR proteins is complicated. CaLRR1 in pepper, for instance, is localized to the cytoplasm and plasma membrane and can be secreted into the apoplastic space [30]. OsLRR1 is localized to the endosomal pathway [9]. These proteins may also be localized to the endoplasmic reticulum [28,56] or extracellular space [61]. Like the subcellular localization of NtLRR2 [29] and CaLRR1 [30], both of which play an important role in the resistance of plants to pathogens, OsI-BAK1 is localized to plasma membranes (Figure 1c). This result demonstrates that OsI-BAK1, in addition to its functions in grain filling, leaf development [43] and herbivore resistance reported in this study, may also play a role in plants’ resistance to disease. This hypothesis needs to be confirmed in the future.

Both MPKs and WRKY transcription factors play a central role in herbivore-induced plant defense responses via modulating defensive signaling pathways, such as those mediated by JA, SA and ethylene [47,62]. Moreover, MPKs and WRKY transcription factors can regulate each other at the transcriptional and translational levels [34,48]. In this study, although silencing *OsI-BAK1* increased the transcript level of *OsMPK3*, it did not influence the activity of OsMPK3 or the transcript level and activity of OsMPK6 (Figure 4a and Figure S5). However, we did observe that silencing *OsI-BAK1* upregulated the transcript levels of *OsWRKY24*, *OsWRKY53* and *OsWRKY70* (Figure 4b–d). Moreover, silencing *OsI-BAK1* decreased the BPH-induced levels of ET (Figure 5a) but not JA, JA-Ile, ABA, SA and H_2_O_2_ (Figure S6). Given that both OsWRKY53 and OsWRKY70 regulate herbivore-induced levels of ET in rice [33,34,48], we think that the decrease in BPH-induced ET levels in ir-*ibak1* plants is probably related to the change in transcript levels of *OsWRKY53* and *OsWRKY70*. Why the levels of these WRKY transcripts and of ET in ir-*ibak1* plants changed bears further study.

The ET-mediated signaling pathway plays an important role in the resistance of plants to herbivores [1,6]. In rice, it has been reported that the ET pathway negatively regulates the resistance of plants to BPH [36]: BPH preferred to feed and oviposit on the WT plants over plants with ET deficiency; moreover, in BPH, the levels of honeydew excretion were reduced, and in plants with ET deficiency, natural enemies became more attractive. Here, we observed a similar result: BPH preferred to oviposit on WT plants over ir-*ibak1* plants. In addition, the exogenous application of ethephon on the ir-*ibak1* lines abolished the difference in BPH oviposition preference (Figure 7), suggesting that the decreased oviposition preference of BPH for ir-*ibak1* plants depends on the ET pathway (as we found previously) [36]. Interestingly, we did not find that the exogenous application of ethephon on ir-*ibak1* plants restored the hatching rate of BPH eggs (Figure S7), demonstrating that other signaling pathways or components regulated the survival of BPH eggs laid on ir-*ibak1* plants. It will be interesting to identify these pathways or components in the future.

ET has been reported to negatively regulate root growth [63]. Moreover, in rice, the overexpression of *OsERF2* reduces the root length [53]. The root length in ir-*ibak1* plants was longer and the levels of ET and *OsERF2* transcripts were lower than in WT plants. Thus, the longer root length phenotype exhibited in ir-*ibak1* plants compared to WT plants may also be related to the impaired ET signaling pathway. Whether other components are involved in this process remains to be elucidated.

In summary, our results show that a plasma-membrane-localized simple eLRR protein, OsI-BAK1, functions as a negative regulator in the defense response of rice to BPH. BPH can benefit by exploiting this regulator, i.e., inducing the expression of *OsI-BAK1*, which then decreases the transcript levels of *OsMPK3*, *OsWRKY24*, *OsWRKY53* and *OsWRKY70* and increases the BPH-induced ET levels, changes that promote a susceptibility to BPH in the host plant. These findings demonstrate how BPH regulates host plant resistance, namely by influencing an early signaling factor.

## 4. Materials and Methods

### 4.1. Plant Growth and Insects

In this study, the rice (*Oryza sativa* L.) genotypes were Xiushui 11 (wild-type, WT) and transgenic lines with silenced *OsI-BAK1* (ir-*ibak1* lines) (see below). Pregerminated seeds of different lines were cultured in plastic culture dishes (diameter 90 mm, height 15 mm) in an incubator at 28 ± 2 °C with a 14-h light period. Ten-day-old seedlings were transferred to hydroponic boxes (20 L) containing a rice nutrient solution [64] and kept in a greenhouse (28 ± 2 °C, 14-h light, 55–65% relative humidity). Twenty to twenty-five days later, plants were transplanted into individual 300-mL hydroponic plastic pots containing a nutrient solution (Figure S1). Plants were used for different experiments 4 d after transplantation. Colonies of BPH and white-backed planthopper (WBPH) *Sogatella furcifera* were originally collected from rice fields in Hangzhou, China and maintained on plants of TN1, a susceptible rice variety, for more than 10 years in a climate chamber (26 ± 2 °C, 14-h light, 65% relative humidity).

### 4.2. Isolation of OsI-BAK1 cDNA

The full-length cDNA of *OsI-BAK1* was obtained by reverse transcription-polymerase chain reaction (RT-PCR) from the total RNA isolated from WT plants. Specific primers OsI-BAK1-F (5′-GACTGCCAGAGCCTCTACCT-3′) and OsI-BAK1-R (5′-GTTCATGCTGCCTGGGTACA-3′) were designed based on the sequence of *OsI-BAK1* (TIGR ID: LOC_Os03g32580). The PCR products were cloned into the pEASY^®^-Blunt simple cloning vector (TransGen, Beijing, China) and sequenced.

### 4.3. Structure and Phylogenetic Analysis of OsI-BAK1

The structure domain of OsI-BAK1 was analyzed using SMART (http://smart.embl.de/; accessed on 4 January 2020) and the MEME Suite (http://meme-suite.org/; accessed on 4 January 2020). The prediction of the signal peptides was performed using SIGNALP 5.0 (http://www.cbs.dtu.dk/services/SignalP/; accessed on 4 January 2020).

The homologs of OsI-BAK1 from other plant species were identified using BLASTP in the NCBI website (https://blast.ncbi.nlm.nih.gov/Blast.cgi; accessed on 10 September 2021). The full-length amino acid sequences of OsI-BAK1 were set as query sequences and blasted with the default parameters. The amino acid sequences were downloaded from the website and aligned by ClustalW in MEGA-X (pairwise alignment: gap opening penalty 10.00, gap extension penalty 0.10; multiple alignment: gap opening penalty 10.00, gap extension penalty 0.20; use negative matrix: off, delay divergent cutoff 30%) [65]. The alignment results were used to construct a neighbor-joining tree with default parameters similar to a previous study (scope: all selected taxa; statistical method: neighbor-joining; test of phylogeny: bootstrap method, 1000 times of replications; substitutions type: amino acid; model/method: Poisson model; rates among sites: uniform rates; pattern among lineages: same (homogeneous); gaps/missing date treatment: complete deletion; number of threads 3) [22].

### 4.4. Subcellular Localization Assay

For subcellular localization, the full length of *OsI-BAK1* was inserted into pCAMBIA1301 and fused with green fluorescent protein (GFP), yielding a transformation plasmid vector (Figure S2a). Protoplast isolation and polyethylene glycol (PEG)-mediated transformation was performed following a method described previously [66]. Briefly, rice protoplasts were prepared from leaf sheaths of seedlings of rice variety *Nipponbare* at a concentration of 2 × 10^6^ cells/mL. Ten micrograms of plasmid DNA (pCAMBIA1301-OsI-BAK1-GFP) was mixed with 100 μL of protoplasts and 110 μL of 40% PEG–calcium transfection solution (40% (*w*/*v*) PEG4000 in ddH_2_O containing 0.2 M of mannitol and 100 mM of CaCl_2_ for 5 min. The reaction was stopped by adding 400 μL of W5 solution (0.1% glucose, 0.08% KCl, 0.9% NaCl, 1.84% CaCl_2_∙2H_2_O and 2-mM MES∙KOH (pH 5.7)). The solution was centrifuged, the supernatant was removed and the remainder was incubated in the dark for 16–20 h at 26 °C. Transformed protoplasts were imaged under a confocal microscope (LSM 800, Zeiss, Oberkochen, Germany). The plasmid pBIN-*AtPIP2A*-*mCherry* [22] was used as a positive control for membrane localization. The chemical compounds mentioned above were all obtained from Sigma-Aldrich (Darmstadt, Germany).

### 4.5. Plant Treatment

For mechanical wounding, plant shoots (lower part, approximately 2 cm long) were individually pierced 200 times with a needle. Nonmanipulated plants were used as controls. For BPH or WBPH treatment, plant shoots were individually infested with 15 gravid females of BPH or WBPH, each of which was confined in a glass cage (diameter 40 mm, height 80 mm, with 24 small holes, diameter 0.8 mm) (Figure S1). Plants with empty cages were used as controls. For the MeJA (Sigma-Aldrich, Darmstadt, Germany) or ABA (Aladdin, Shanghai, China) treatment, according to our previous experiments [67], MeJA or ABA was first dissolved in a small volume of ethanol (100%) and then added to the nutrient solution until its concentration reached 100-μM. Plants grown in the nutrient solution with an equal volume of ethanol (100%) but without MeJA or ABA were used as controls. For ethephon treatment, ethephon (Aladdin, Shanghai, China) was dissolved in ddH_2_O (with 0.02% Tween-20), and then, each plant was sprayed thoroughly with 500-μM or 1-mM ethephon solution. Controls were sprayed with equal volumes of 0.02% Tween-20 in ddH_2_O.

### 4.6. RNA Extraction and Real Time-qPCR

In these experiments, five independent biological samples were used. Total RNA was extracted from 100 mg of rice leaf sheaths using the MiniBEST Plant RNA Extraction Kit (TaKaRa, Dalian, China). Five hundred nanograms of each total RNA sample was reverse-transcribed by using PrimeScript™ RT Master Mix (TaKaRa, Dalian, China) according to the manufacturer’s protocols. The real-time qPCR assay was performed on the CFX96 Real-Time System (Bio-Rad, Richmond, CA, USA), using TB Green^®^ Premix Ex Taq™ II or Premix Ex Taq™ (Probe qPCR) (TaKaRa, Dalian, China) following the manufacturer’s protocols. A linear standard curve, threshold cycle number versus log_10_ (designated transcript level), was built using a series of concentrations of a specific cDNA standard, and the relative expression levels of the transcripts of different genes were determined according to the standard curve. A rice actin gene, *OsACTIN* (TIGR ID: LOC_Os03g50885), was used as an internal standard to normalize the cDNA concentrations. All primers and probes of the target genes used for real-time qPCR are listed in Table S1.

### 4.7. Generation and Characterization of Transgenic Plants

To prepare the RNAi construct, a 520-bp fragment of *OsI-BAK1* was cloned into the pCAMBIA1301-RNAi vector (Figure S2b). This vector was inserted into rice variety Xiushui 11 using *Agrobacterium tumefaciens*-mediated transformation. Rice transformation, screening of the homozygous T_2_ plants and identification of the number of insertions by Southern blot were performed following a method described previously [38]. The silencing efficacy of *OsI-BAK1* in the ir-*ibak1* lines was performed using real-time qPCR with primers irIBAK1-F (5′-GGCCTTCACCCTCTTCTACT-3′) and irIBAK1-R (5′-GTCGTAGGTAGAGGCTCTGG-3′). Two homozygous T_2_ lines (irIBAK1-1 and irIBAK1-2) with a single insertion were selected and used in the following experiments.

### 4.8. Measurement of Plant Growth Parameters

Plant growth parameters of the 30- and 40-day-old WT plants and of the ir-*ibak1* plants were measured. These plant growth parameters included the root length, shoot height, plant mass and chlorophyll content. The root length was measured from the shoot base to the longest root tip. The shoot height was measured from the shoot base to the longest leaf apex. The whole weight of the plant was regarded as the plant mass. For the chlorophyll content determination, the youngest three fully expanded leaves from each plant and three locations (at the tip, middle and base of the leaf) from each leaf were measured by a chlorophyll meter, SPAD-502 Plus (Tokyo, Japan). The experiments above were replicated 10 times.

### 4.9. BPH Bioassay

To investigate the effect of *OsI-BAK1* on the hatching rate of the BPH eggs, the WT and ir-*ibak1* plants were individually confined in glass cages, and 15 gravid BPH females were introduced into each cage (Figure S1). Twelve hours later, the females were removed, and the number of newly hatched nymphs on each plant was counted every day until no newborn nymphs appeared for three consecutive days. The unhatched eggs in each plant were counted under a microscope to calculate the hatching rate. The experiments for each line were replicated 10 times.

To assess the influence of silencing *OsI-BAK1* on the survival rate of BPH nymphs, the WT and ir-*ibak1* plants were individually confined in glass cages, and 15 newly hatched BPH nymphs were released into each cage (Figure S1). The surviving nymphs on each plant were recorded daily until all of them became adults. The experiments for each line were replicated 10 times.

To observe the impact of *OsI-BAK1* on the BPH oviposition preference, pots with two plants—an irIBAK1-1 plant and a WT plant or an irIBAK1-2 plant and a WT plant—were individually confined in glass cages (see Figure S1 but with two plants). Fifteen gravid BPH females were released into each cage. Two days later, the insects were removed, and the number of eggs on each plant was counted under a microscope. The experiment was replicated 10 times.

### 4.10. Detection of MPK Activation

Plants of the WT and two ir-*ibak1* lines were randomly assigned to BPH and control treatments. The outermost part of two leaf sheaths that were infested by gravid BPH females was harvested at 0, 1 and 3 h after BPH infestation. The experiments for each treatment at each time point were replicated five times. The sample containing five replications was mixed and ground together, and 100 mg of each sample was used to extract the total proteins and detect the MPK activation according to a method described previously [39]. The quantitative results were calculated after repeating this experiment for four times using ImageJ (https://imagej.nih.gov/ij/; accessed on 16 July 2021). The relative kinase activation of OsMPK3 and OsMPK6 was calculated by taking the signal intensity of the WT sample without BPH infestation (at 0 h) as 1 for normalization.

### 4.11. SA, JA, JA-Ile, ABA and H_2_O_2_ Analysis

Plants of the WT and two transgenic lines were randomly assigned to BPH and control treatments. The outermost part of two leaf sheaths that were infested by gravid BPH females was harvested at 0, 8, 24 and 48 h after BPH infestation. The SA, JA, JA-Ile and ABA were extracted with ethyl acetate spiked with labeled internal standards (^2^D6-JA, ^2^D6-JA-Ile, ^2^D4-SA and ^2^D6-ABA) and analyzed using a HPLC/mass spectrometry/mass spectrometry system [22]. For the H_2_O_2_ analysis, the outermost part of two leaf sheaths was collected at 0, 8, 12 and 24 h after BPH infestation. The H_2_O_2_ concentration of each sample was measured using the Amplex Red Hydrogen Peroxide/Peroxidase Assay Kit (Invitrogen, Eugene, OR, USA) according to the instructions. The experiments for each treatment at each time point were replicated five times.

### 4.12. Ethylene Analysis

Plants of the WT and two ir-*ibak1* lines, each with 15 gravid BPH females, were individually covered in sealed glass cylinders (diameter 4 cm, height 50 cm). ET production was measured at 24, 48 and 72 h after BPH infestation by gas chromatography according to the method [36]. The experiments for each line at each time point were replicated eight times.

### 4.13. Data Analysis

Two-treatment data were analyzed using Student’s *t*-tests. Data from three or more treatments were compared using one-way ANOVA followed by Tukey’s HSD post-hoc tests. All tests were carried out with SPSS software version 18 (IBM Corp., Armonk, NY, USA).

## Data Availability

Not applicable.

## Supplementary Materials

The following are available online at www.mdpi.com/1422-0067/222/21/2182/s1.

## References

[1] Erb M., Reymond P. (2019). Molecular interactions between plants and insect herbivores. Annu. Rev. Plant Biol..

[2] Wu J., Baldwin I.T. (2010). New insights into plant responses to the attack from insect herbivores. Annu. Rev. Genet..

[3] Arimura G., Ozawa R., Maffei M.E. (2011). Recent advances in plant early signaling in response to herbivory. Int. J. Mol. Sci..

[4] Furstenberg-Hagg J., Zagrobelny M., Bak S. (2013). Plant defense against insect herbivores. Int. J. Mol. Sci..

[5] Schuman M.C., Baldwin I.T. (2016). The layers of plant responses to insect herbivores. Annu. Rev. Entomol..

[6] Stahl E., Hilfiker O., Reymond P. (2018). Plant-arthropod interactions: Who is the winner?. Plant J..

[7] Park Y.H., Choi C., Park E.M., Kim H.S., Park H.J., Bae S.C., Ahn I., Kim M.G., Park S.R., Hwang D.J. (2012). Over-expression of rice leucine-rich repeat protein results in activation of defense response, thereby enhancing resistance to bacterial soft rot in Chinese cabbage. Plant Cell Rep..

[8] Van der Hoorn R.A.L., Wulff B.B.H., Rivas S., Durrant M.C., van der Ploeg A., de Wit P.J.G.M., Jones J.D.G. (2005). Structure‒function analysis of Cf-9, a receptor-like protein with extracytoplasmic leucine-rich repeats. Plant Cell.

[9] Zhou L., Cheung M.Y., Zhang Q., Lei C.L., Zhang S.H., Sun S.S., Lam H.M. (2009). A novel simple extracellular leucine-rich repeat (eLRR) domain protein from rice (OsLRR1) enters the endosomal pathway and interacts with the hypersensitive-induced reaction protein 1 (OsHIR1). Plant Cell Environ..

[10] Couto D., Zipfel C. (2016). Regulation of pattern recognition receptor signalling in plants. Nat. Rev. Immunol..

[11] Van der Burgh A.M., Joosten M. (2019). Plant immunity: Thinking outside and inside the box. Trends Plant Sci..

[12] Wang W., Feng B., Zhou J.M., Tang D. (2020). Plant immune signaling: Advancing on two frontiers. J. Integr. Plant Biol..

[13] Chinchilla D., Bauer Z., Regenass M., Boller T., Felix G. (2006). The *Arabidopsis* receptor kinase FLS2 binds flg22 and determines the specificity of flagellin perception. Plant Cell.

[14] Gómez-Gómez L., Boller T. (2000). FLS2: An LRR receptor‒like kinase involved in the perception of the bacterial elicitor flagellin in *Arabidopsis*. Mol. Cell.

[15] Sun Y., Li L., Macho A.P., Han Z., Hu Z., Zipfel C., Zhou J.M., Chai J. (2013). Structural basis for flg22-induced activation of the *Arabidopsis* FLS2-BAK1 immune complex. Science.

[16] Mithoe S.C., Menke F.L. (2018). Regulation of pattern recognition receptor signalling by phosphorylation and ubiquitination. Curr. Opin. Plant Biol..

[17] Zhou Z., Zhao Y., Bi G., Liang X., Zhou J.M. (2019). Early signalling mechanisms underlying receptor kinase-mediated immunity in plants. Philos. Trans. R. Soc. Lond. B Biol. Sci..

[18] Luu D.D., Joe A., Chen Y., Parys K., Bahar O., Pruitt R., Chan L.J.G., Petzold C.J., Long K., Adamchak C. (2019). Biosynthesis and secretion of the microbial sulfated peptide RaxX and binding to the rice XA21 immune receptor. Proc. Natl. Acad. Sci. USA.

[19] Pruitt R.N., Schwessinger B., Joe A., Thomas N., Liu F., Albert M., Robinson M.R., Chan L.J., Luu D.D., Chen H. (2015). The rice immune receptor XA21 recognizes a tyrosine-sulfated protein from a Gram-negative bacterium. Sci. Adv..

[20] Song W., Wang G., Chen L., Kim H.S., Pi L., Holsten T., Gardner J., Wang B., Zhai W., Zhu L. (1995). A receptor kinase-like protein encoded by the rice disease resistance gene, *Xa21*. Science.

[21] Wang G.L., Song W.Y., Ruan D.L., Sideris S., Ronald P.C. (1996). The cloned gene, *Xa21*, confers resistance to multiple *Xanthomonas oryzae* pv. *oryzae* isolates in transgenic plants. Mol. Plant. Microbe Interact..

[22] Hu L., Ye M., Kuai P., Ye M., Erb M., Lou Y. (2018). OsLRR-RLK1, an early responsive leucine-rich repeat receptor-like kinase, initiates rice defense responses against a chewing herbivore. New Phytol..

[23] Steinbrenner A.D., Munoz-Amatriain M., Chaparro A.F., Aguilar-Venegas J.M., Lo S., Okuda S., Glauser G., Dongiovanni J., Shi D., Hall M. (2020). A receptor-like protein mediates plant immune responses to herbivore-associated molecular patterns. Proc. Natl. Acad. Sci. USA.

[24] Kirsch R., Vurmaz E., Schaefer C., Eberl F., Sporer T., Haeger W., Pauchet Y. (2020). Plants use identical inhibitors to protect their cell wall pectin against microbes and insects. Ecol. Evol..

[25] Tornero P., Mayda E., Gomez M.D., Canas L., Conejero V., Vera P. (1996). Characterization of LRP, a leucine-rich repeat (LRR) protein from tomato plants that is processed during pathogenesis. Plant J..

[26] Hipskind J.D., Nicholson R.L., Goldsbrough P.B. (1996). Isolation of a cDNA encoding a novel leucine-rich repeat motif from *Sorghum bicolor* inoculated with fungi. Mol. Plant. Microbe Interact..

[27] Zhu F.-Y., Li L., Zhang J., Lo C. (2015). Transgenic expression of a sorghum gene (*SbLRR2*) encoding a simple extracellular leucine-rich protein enhances resistance against necrotrophic pathogens in *Arabidopsis*. Physiol. Mol. Plant Pathol..

[28] Jacques A., Ghannam A., Erhardt M., de Ruffray P., Baillieul F., Kauffmann S. (2006). *NtLRP1*, a tobacco leucine-rich repeat gene with a possible role as a modulator of the hypersensitive response. Mol. Plant. Microbe Interact..

[29] Xu Z., Xiong T., Ni Z., Chen X., Chen M., Li L., Gao D., Yu X., Liu P., Ma Y. (2009). Isolation and identification of two genes encoding leucine-rich repeat (LRR) proteins differentially responsive to pathogen attack and salt stress in tobacco. Plant Sci..

[30] Hong J.K., Hwang I.S., Hwang B.K. (2017). Functional roles of the pepper leucine-rich repeat protein and its interactions with pathogenesis-related and hypersensitive-induced proteins in plant cell death and immunity. Planta.

[31] Caddell D.F., Park C.J., Thomas N.C., Canlas P.E., Ronald P.C. (2017). Silencing of the rice gene *LRR1* compromises rice *Xa21* transcript accumulation and XA21-mediated immunity. Rice.

[32] Lou Y., Zhang G., Zhang W., Hu Y., Zhang J. (2013). Biological control of rice insect pests in China. Biol. Control.

[33] Hu L., Ye M., Li R., Lou Y. (2016). OsWRKY53, a versatile switch in regulating herbivore-induced defense responses in rice. Plant Plant Signal. Behav..

[34] Li R., Zhang J., Li J., Zhou G., Wang Q., Bian W., Erb M., Lou Y. (2015). Prioritizing plant defence over growth through WRKY regulation facilitates infestation by non-target herbivores. eLife.

[35] Lu J., Ju H., Zhou G., Zhu C., Erb M., Wang X., Wang P., Lou Y. (2011). An EAR-motif-containing ERF transcription factor affects herbivore-induced signaling, defense and resistance in rice. Plant J..

[36] Lu J., Li J., Ju H., Liu X., Erb M., Wang X., Lou Y. (2014). Contrasting effects of ethylene biosynthesis on induced plant resistance against a chewing and a piercing-sucking herbivore in rice. Mol. Plant.

[37] Zheng X., Zhu L., He G. (2021). Genetic and molecular understanding of host rice resistance and *Nilaparvata lugens* adaptation. Curr. Opin. Insect Sci..

[38] Zhou G., Qi J., Ren N., Cheng J., Erb M., Mao B., Lou Y. (2009). Silencing *OsHI-LOX* makes rice more susceptible to chewing herbivores, but enhances resistance to a phloem feeder. Plant J..

[39] Ye M., Kuai P., Hu L., Ye M., Sun H., Erb M., Lou Y. (2020). Suppression of a leucine-rich repeat receptor-like kinase enhances host plant resistance to a specialist herbivore. Plant Cell Environ..

[40] Zhang J., Luo T., Wang W., Cao T., Li R., Lou Y. (2017). Silencing *OsSLR1* enhances the resistance of rice to the brown planthopper *Nilaparvata lugens*. Plant Cell Environ..

[41] Xu J., Wang X., Zu H., Zeng X., Baldwin I.T., Lou Y., Li R. (2021). Molecular dissection of rice phytohormone signaling involved in resistance to a piercing-sucking herbivore. New Phytol..

[42] Zeng J., Zhang T., Huangfu J., Li R., Lou Y. (2021). Both allene oxide synthases genes are involved in the biosynthesis of herbivore-induced jasmonic acid and herbivore resistance in rice. Plants.

[43] Khew C.Y., Teo C.J., Chan W.S., Wong H.L., Namasivayam P., Ho C.L. (2015). Brassinosteroid insensitive 1-associated kinase 1 (OsI-BAK1) is associated with grain filling and leaf development in rice. J. Plant Physiol..

[44] Viana V.E., Busanello C., da Maia L.C., Pegoraro C., de Oliveira A.C. (2018). Activation of rice WRKY transcription factors: An army of stress fighting soldiers?. Curr. Opin. Plant Biol..

[45] Zhang M., Su J., Zhang Y., Xu J., Zhang S. (2018). Conveying endogenous and exogenous signals: MAPK cascades in plant growth and defense. Curr. Opin. Plant Biol..

[46] Shen X., Yuan B., Liu H., Li X., Xu C., Wang S. (2010). Opposite functions of a rice mitogen-activated protein kinase during the process of resistance against *Xanthomonas oryzae*. Plant J..

[47] Wang Q., Li J., Hu L., Zhang T., Zhang G., Lou Y. (2013). *OsMPK3* positively regulates the JA signaling pathway and plant resistance to a chewing herbivore in rice. Plant Cell Rep..

[48] Hu L., Ye M., Li R., Zhang T., Zhou G., Wang Q., Lu J., Lou Y. (2015). The rice transcription factor WRKY53 suppresses herbivore-induced defenses by acting as a negative feedback modulator of mitogen-activated protein kinase activity. Plant Physiol..

[49] Yokotani N., Shikata M., Ichikawa H., Mitsuda N., Ohme-Takagi M., Minami E., Nishizawa Y. (2018). OsWRKY24, a blast-disease responsive transcription factor, positively regulates rice disease resistance. J. Gen. Plant Pathol..

[50] Zhou G., Ren N., Qi J., Lu J., Xiang C., Ju H., Cheng J., Lou Y. (2014). The 9-lipoxygenase Osr9-LOX1 interacts with the 13-lipoxygenase-mediated pathway to regulate resistance to chewing and piercing-sucking herbivores in rice. Physiol. Plant.

[51] Guo Y., Zhu C., Gan L., Ng D., Xia K. (2014). Ethylene is involved in the complete-submergence induced increase in root iron and manganese plaques in *Oryza sativa*. Plant Growth Regul..

[52] Rzewuski G., Sauter M. (2008). Ethylene biosynthesis and signaling in rice. Plant Sci..

[53] Xiao G., Qin H., Zhou J., Quan R., Lu X., Huang R., Zhang H. (2016). OsERF2 controls rice root growth and hormone responses through tuning expression of key genes involved in hormone signaling and sucrose metabolism. Plant Mol. Biol..

[54] Zhou L., Cheung M.Y., Li M.W., Fu Y., Sun Z., Sun S.M., Lam H.M. (2010). Rice hypersensitive induced reaction protein 1 (OsHIR1) associates with plasma membrane and triggers hypersensitive cell death. BMC Plant Biol..

[55] Jung E.H., Jung H.W., Lee S.C., Han S.W., Heu S., Hwang B.K. (2004). Identification of a novel pathogen-induced gene encoding a leucine-rich repeat protein expressed in phloem cells of *Capsicum annuum*. Biochim. Biophys. Acta.

[56] Ravindran P., Yong S.Y., Mohanty B., Kumar P.P. (2020). An LRR-only protein regulates abscisic acid-mediated abiotic stress responses during *Arabidopsis* seed germination. Plant Cell Rep..

[57] Lee D.H., Lee H.S., Belkhadir Y. (2021). Coding of plant immune signals by surface receptors. Curr. Opin. Plant Biol..

[58] Reymond P. (2021). Receptor kinases in plant responses to herbivory. Curr. Opin. Biotechnol..

[59] Herger A., Dunser K., Kleine-Vehn J., Ringli C. (2019). Leucine-rich repeat extensin proteins and their role in cell wall sensing. Curr. Biol..

[60] Kalunke R.M., Tundo S., Benedetti M., Cervone F., De Lorenzo G., D′Ovidio R. (2015). An update on polygalacturonase-inhibiting protein (PGIP), a leucine-rich repeat protein that protects crop plants against pathogens. Front. Plant Sci..

[61] Zhu F., Li L., Lam P., Chen M., Chye M., Lo C. (2013). Sorghum extracellular leucine-rich repeat protein SbLRR2 mediates lead tolerance in transgenic Arabidopsis. Plant Cell Physiol..

[62] Huangfu J., Li J., Li R., Ye M., Kuai P., Zhang T., Lou Y. (2016). The Transcription factor OsWRKY45 negatively modulates the resistance of rice to the brown planthopper *Nilaparvata lugens*. Int. J. Mol. Sci..

[63] Yang C., Lu X., Ma B., Chen S.Y., Zhang J.S. (2015). Ethylene signaling in rice and *Arabidopsis*: Conserved and diverged aspects. Mol. Plant.

[64] Yoshida S., Forno D.A., Cock J.H. (1976). Laboratory Manual for Physiological Studies of Rice.

[65] Kumar S., Stecher G., Li M., Knyaz C., Tamura K. (2018). MEGA X: Molecular evolutionary genetics analysis across computing platforms. Mol. Biol. Evol..

[66] You Q., Zhai K., Yang D., Yang W., Wu J., Liu J., Pan W., Wang J., Zhu X., Jian Y. (2016). An E3 ubiquitin ligase-BAG protein module controls plant innate immunity and broad-spectrum disease resistance. Cell Host Microbe.

[67] Zhou S., Chen M., Zhang Y., Gao Q., Noman A., Wang Q., Li H., Chen L., Zhou P., Lu J. (2019). *OsMKK3*, a stress-responsive protein kinase, positively regulates rice resistance to *Nilaparvata lugens* via phytohormone dynamics. Int. J. Mol. Sci..

