# Supplementary material for: Silencing a Simple Extracellular Leucine-Rich Repeat Gene OsI-BAK1 Enhances the Resistance of Rice to Brown Planthopper Nilaparvata lugens
Source: Int J Mol Sci. 2021 Nov 10;22(22):12182. doi: 10.3390/ijms222212182 (PMC8622231; doi:10.3390/ijms222212182)
Supplement: Supplementary file 1 [file ijms-22-12182-s001.zip › ijms-1447770-supplementary.pdf]

Supplementary Materials

Silencing a Simple Extracellular Leucine-Rich Repeat Gene *OsI-BAK1* Enhances the Resistance of Rice to Brown Planthopper *Nilaparvata lugens*

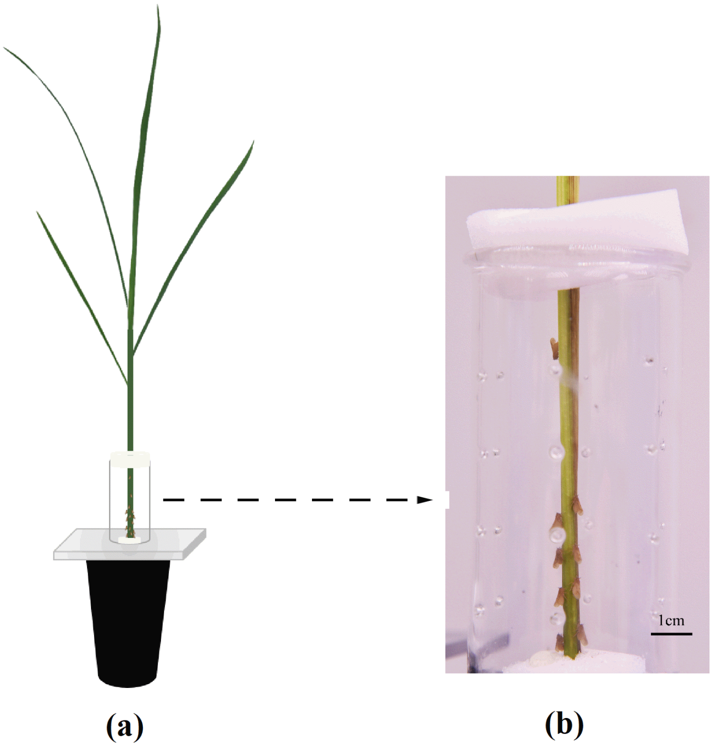

Figure S1. Setups for herbivore bioassay or treatment.

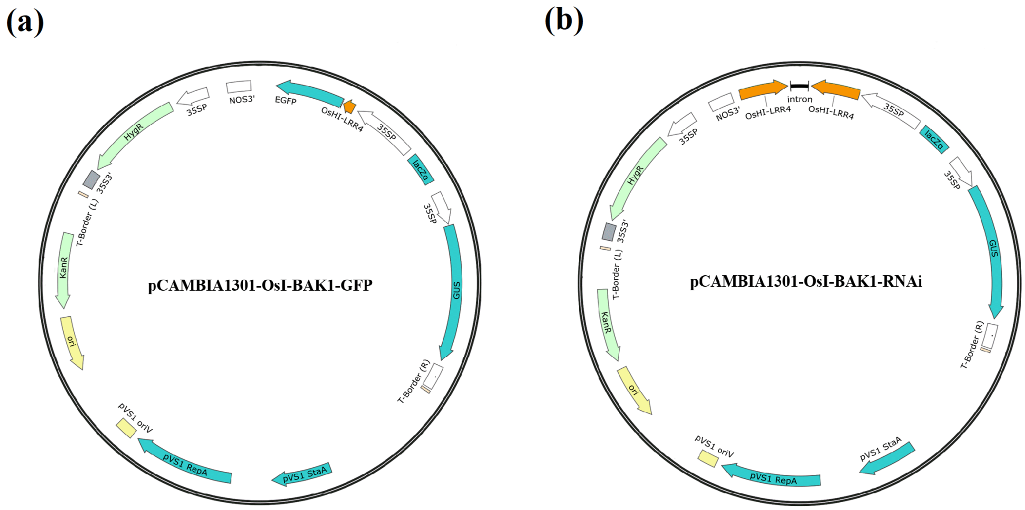

Figure S2. Vectors used in this study. (**a**) The vector used for subcellular localization of *OsI-BAK1*. (**b**) The vector used for obtaining transgenic plants by silencing *OsI-BAK1*.

```
1          ATGGCGCCCCGCGTGTCCCTGCTCGCCGCCGGCGCCGTGGCCGTGGCCGTGGTCCTGCTT
1           M  A  P  R  V  S  L  L  A  A  G  A  V  A  V  A  V  V  L  L

61         CTCGCCGCGCCGGCGCGGGCGTCCAACGACGAGGGGGACGCGCTGTACGCGCTGCGGACG
21          L  A  A  P  A  R  A  S  N  D  E  G  D  A  L  Y  A  L  R  T

121        AGGCTGTCGGATCCCAACGGCGTGCTGCAGAGCTGGGACCCGACCCTCGTCAACCCCTGC
41          R  L  S  D  P  N  G  V  L  Q  S  W  D  P  T  L  V  N  P  C

181        ACCTGGTTCCATGTCACCTGCGACCACGCCAGCCGCGTCGTCCGCCTGGATTTAGGAAAC
61          T  W  F  H  V  T  C  D  H  A  S  R  V  V  R  L  D  L  G  N

241        TCCAACATCTCCGGCTCGATTGGCCCTGAGCTAGGCCGTCTTGTGAACCTCCAATACCTG
81          S  N  I  S  G  S  I  G  P  E  L  G  R  L  V  N  L  Q  Y  L

301        GAGCTCTACAGGAACAATCTTAACGGTGAGATCCCAAAAGAATTGGGCAATCTCAAGAAT
101         E  L  Y  R  N  N  L  N  G  E  I  P  K  E  L  G  N  L  K  N

361        TTGATCAGCTTGGATTTGTATGCCAACAAGCTCACTGGAACAATCCCCAAGTCGCTTTCC
121         L  I  S  L  D  L  Y  A  N  K  L  T  G  T  I  P  K  S  L  S

421        AAGCTCGGCTCGCTGAGATTCATGCGGTTGAACAATAACAAGCTTGCTGGATCAATTCCA
141         K  L  G  S  L  R  F  M  R  L  N  N  N  K  L  A  G  S  I  P

481        AGGGAGCTGGCCAAACTATCCAACCTGAAAGTCATTGACTTGTCTAACAATGACCTCTGT
161         R  E  L  A  K  L  S  N  L  K  V  I  D  L  S  N  N  D  L  C

541        GGAACTATTCCTGTTGACGGTCCCTTCTCAACCTTCCCTCTTCGAAGCTTTGAGAACAAC
181         G  T  I  P  V  D  G  P  F  S  T  F  P  L  R  S  F  E  N  N

601        AACAGGCTCAACGGCCCAGAGCTGCAAGGTTTGGTTCCTTATGACTTTGGATGTTAA
201         N  R  L  N  G  P  E  L  Q  G  L  V  P  Y  D  F  G  C  *
```

Figure S3. Sequences of nucleotides and deduced amino acids of OsI-BAK1.

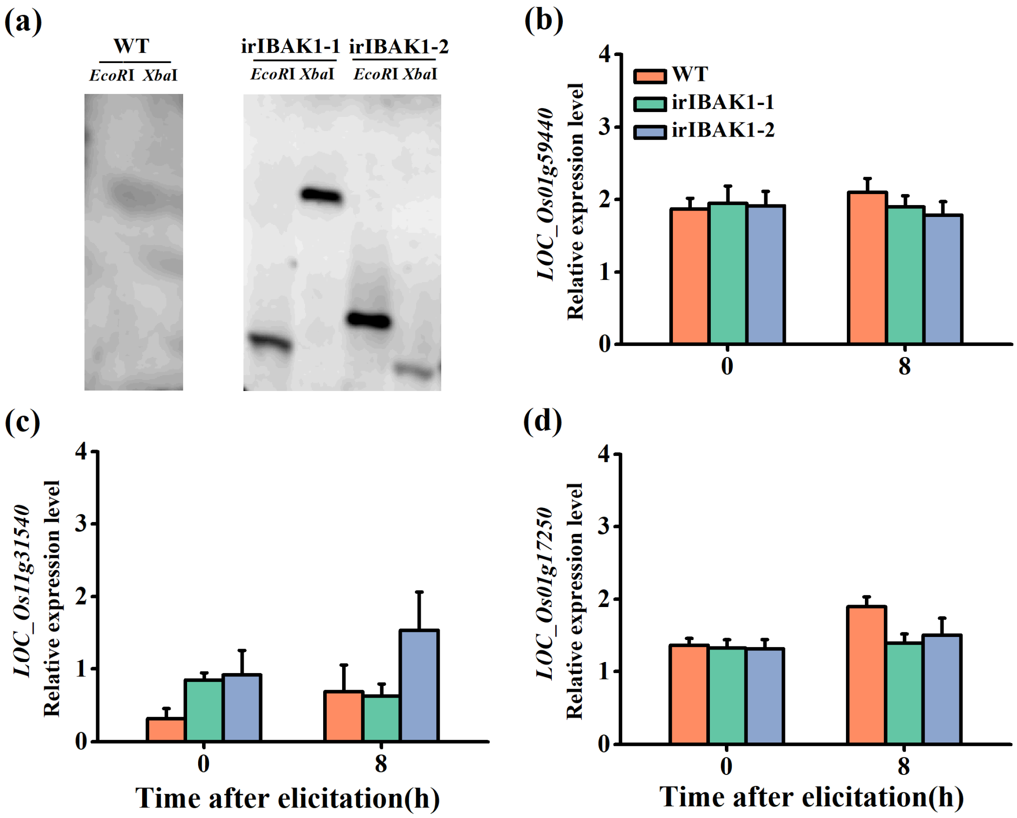

Figure S4. DNA gel-bolt analysis of wild type (WT) and ir-*ibak1* plants and the transcript level of genes sharing high similarity with *OsI-BAK1* in ir-*ibak1* plants. (**a**) Genomic DNA was digested with *Eco*RI or *Xba*I. The blot was hybridized with a probe specific for reporter gene GUS. (**b–d**) Mean expression levels (+ SE, n = 5) of LOC_Os01g59440 (b), LOC_Os11g31540 (c) and LOC_Os01g17250 (d) at 0 and 8 h after BPH infestation.

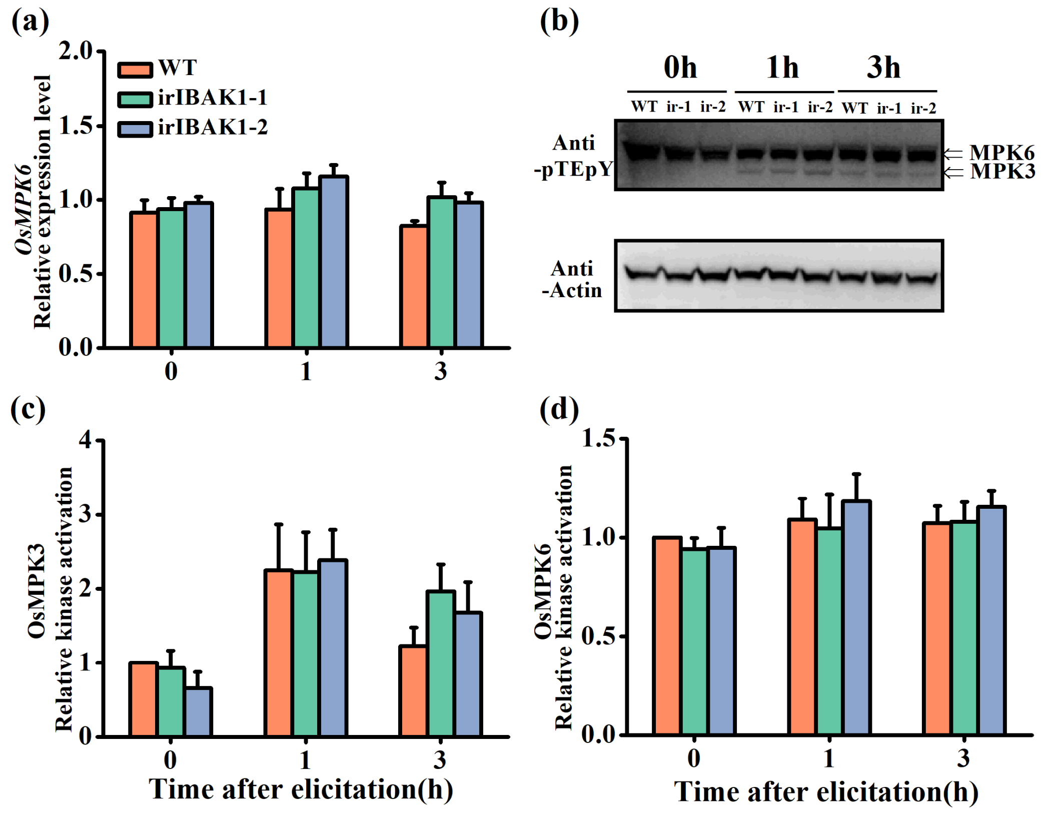

Figure S5. OsI-BAK1 does not affect BPH-induced transcript levels and kinase activation of OsMPK3 and OsMPK6. (**a**) Mean transcript level (+ SE, n = 5) of *OsMPK6* in WT and ir-*ibak1* plants after BPH infestation. (**b**) MPK activity in wild type (WT) and ir-*ibak1* plants at 0, 1 and 3 h after gravid BPH female infestation. ir-1 represents irIBAK1-1; ir-2 represents irIBAK1-2. The rice leaf sheaths from five replicate plants were harvested at the indicated times. Immunoblotting was performed using either anti-pTEpY antibody (first section) to detect phosphorylated MPKs or actin antibodies (second section) as a loading control. This experiment was replicated 4 times with similar results. (c and d) The quantitative relative MPK kinase activity for OsMPK3 (**c**) and OsMPK6 (**d**) versus OsACTIN, respectively. Quantitative analysis (+SE, n = 4) of each line at each time point was calculated taking WT sample without BPH infestation (at 0h) as 1 for normalization.

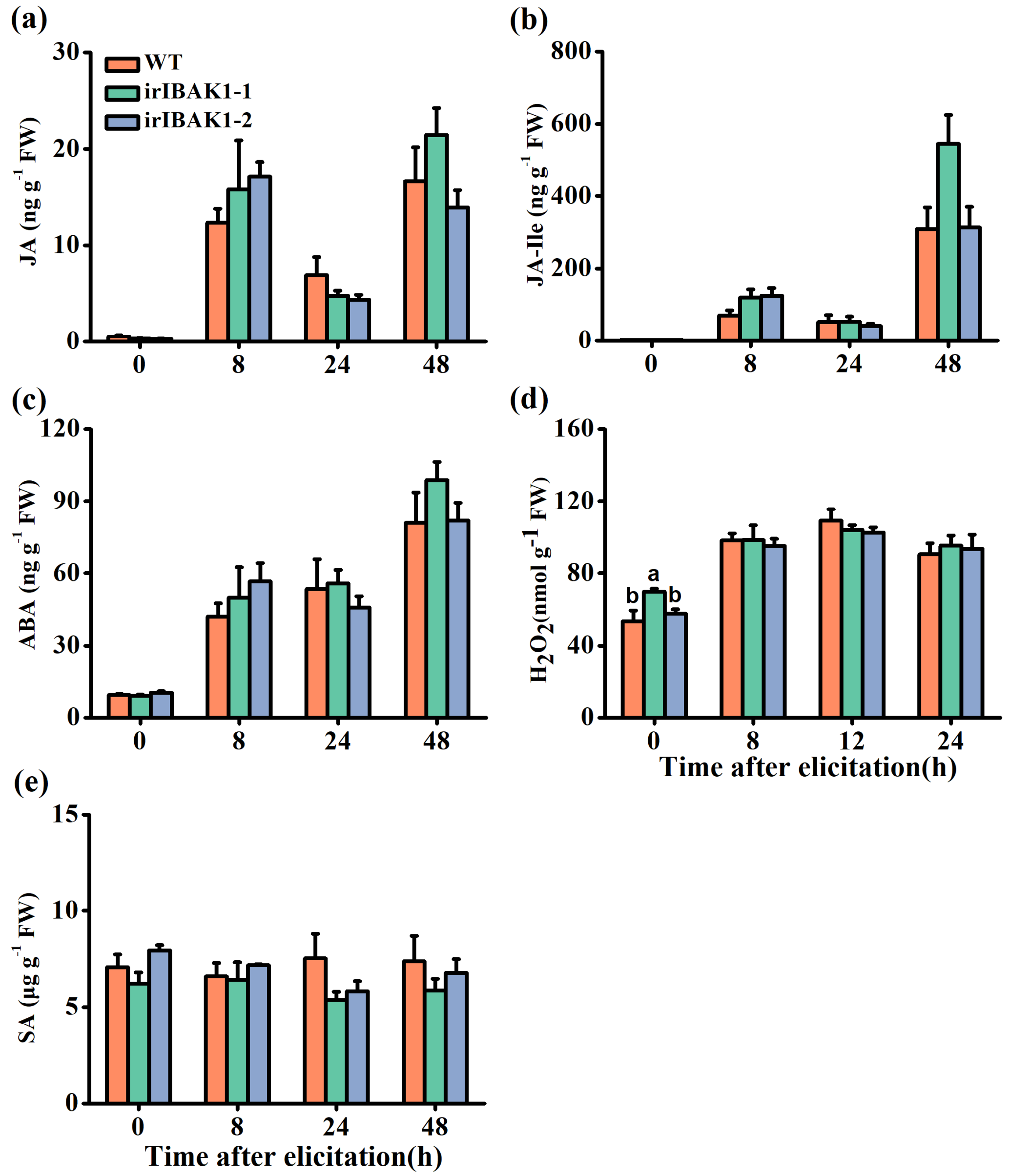

Figure S6. Silencing *OsI-BAK1* does not influence basal and BPH-induced levels of JA, JA-Ile, ABA, H_2O_2 and SA. (**a**–**c**) Mean contents (+ SE, n = 5) of JA (a), JA-Ile (b) and ABA (c) in wild type (WT) and ir-*ibak1* plants at 0, 8, 24 and 48 h after infestation of gravid BPH females. (**d**) Mean contents (+ SE, n = 5) of H_2O_2 in WT and ir-*ibak1* plants at 0, 8, 12 and 24 h after infestation of gravid BPH females. (**e**) Mean contents (+ SE, n = 5) of SA in WT and ir-*ibak1* plants at 0, 8, 24 and 48 h after infestation of gravid BPH females.

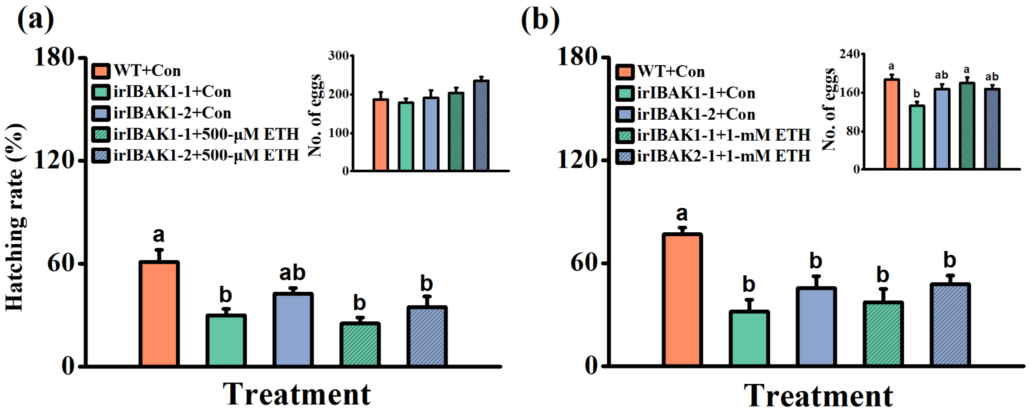

Figure S7. Spraying ethephon does not recover the hatching rate of BPH eggs on ir-*ibak1* plants. (**a**) Mean hatching rate (+ SE, n = 8) of BPH eggs on wild type (WT) and ir-*ibak1* plants after they were sprayed thoroughly with 500-μM ethephon or not. Inserts: Mean numbers of BPH eggs (+ SE, n = 8) on WT and ir-*ibak1* plants. (**b**) Mean hatching rate (+ SE, n = 8) of BPH eggs on WT and ir-*ibak1* plants after they were sprayed thoroughly with 1-mM ethephon or not. Inserts: Mean number of BPH eggs (+ SE, n = 8) on WT and ir-*ibak1* plants. Con, control solution. ETH, ethephon. Different letters represent significant difference among treatments ($p < 0.05$; Tukey's HSD post-doc tests).

Table S1. Primers and probes used for real time-qPCR

Gene names	TIGR ID	Forward primer (5′-…3′)	Reverse primer (5′-…3′)	Probe (5′-…3′)
OsI-BAK1	LOC_Os03g32580	TTGCTGGATCAATTCCAAGG	GAAGGTTGAGAAGGGACCGT	CTGGCCAAACTATCCAACCTGAAAG
OsACTIN	LOC_Os03g50885	TGGACAGGTTATCACCATTGGT	CCGCAGCTTCCATTCCTATG	CGTTTCCGCTGCCCTGAGGTCC
OsWRKY24	LOC_Os01g61080	AAGAGATGGAGGAAAGACGGTG	TGTCGATGTCGCTCATGGTT	AGGGGATCTCCATGGCTGGCAA
OsWRKY53	LOC_Os05g27730	AACGGCTGCTCCATGAAGAA	TTGTGTGCGCCCTTGTAGAC	CTCGCCGACGGCCGCATC
OsWRKY70	LOC_Os05g39720	CCGCTGCTGTTTTGATCATCT	GGAGCTAAGCTAACTCACTCCACA	ATCGGGCCGTCAATTTGATCAGCA
OsMPK3	LOC_Os03g17700	CGACTTCGAGCAGAAGGCTCTA	GTTCATCTCGATCGCTTCGTT	ACGAGGACCAAATGAAGCAGCTGAT
OsMPK6	LOC_Os06g06090	CGCACGCTCAGGGAGATC	GGTATGATATCCCTTATGGCAACAA	CTCCGCCACATGGACCACGAGAA
OsACO1	LOC_Os09g27750	CCAGCTGGTGAAAGAGAGGA	CATGTCGATGATCGGGAACG	
OsACS1	LOC_Os03g51740	GCTCAGGTCTCTCCAGTGTT	ACTTGGCTCACCATCTCCAA	
OsERF2	LOC_Os06g08340	GTGGACCAGATGATCGAGGAG	GATGAACAGGGCACATCAGC	
	LOC_Os01g59440	GCCTGGATCTTGGGAATTT	TTCCGATGGGATCGTTCCTT	
	LOC_Os11g31540	AGTTGGGATCCAACCCTTGT	CCAGCTGTGGAATCAGAGGA	
	LOC_Os01g17250	GAGCTCGGGAGTCTGAAGAA	CCTCGGGATTGGACCGTTTA	
